# Laryngology and Rhinology

**Published:** 1897-12

**Authors:** Barclay J. Baron


### LARYNGOLOGY AND RHINOLOGY.

Gleitsmann[3] draws attention to the influence of **adenoid vegetations** in the naso-pharynx on the growth and configuration of the upper maxilla and the nasal septum. Previous to the second dentition the palate assumes a higher elevation and the alveolar processes approach each other more nearly laterally. The antero-posterior axis of the palate is elongated and the lateral one shortened. The milk teeth, however, are in normal positions. After the second dentition there is exaggeration of these abnormalities and the anterior part of the alveolar process becomes inclined forward, forms an angle at the median junction, and the maxilla becomes V-shaped. If the growth of the bone has been retarded, there is not enough space for the teeth and they are crowded out of their normal place, and the "bite" is not well and evenly made; and to rectify these deformities the aid of the dentist is called for, and "regulators" have to be worn. The effect of this upward pressure of the palate is to cause deflection of the nasal septum. This is rare before the seventh year, but common after the second dentition. The *rôle* of adenoid vegetation in causing these troubles is said to be two-fold: (1) They impede nasal respiration; from this the nasal cavities remain small and ill-developed and the palate becomes elevated. (2) Keeping the mouth open for respiration causes the cheeks to exert pressure against the maxilla laterally. Thus

[3] *J. Laryngol.*, 1897, xii. 357.

the alveolar process is narrowed and lengthened during the milk-teeth stage; and this goes on to a greater extent after the second teeth arrive, and the superior maxilla is bent at its anterior junction and becomes V-shaped. Especially rapid are the changes during the time when the milk teeth are gone and not yet replaced by the permanent ones, and Koerner assumes that they are so, because of the loss of firmness in the maxilla due to the loss of the teeth and also because of an extra influx of blood during the growth of the permanent teeth. The inferior maxilla does not suffer from this pressure because the tongue counteracts it by lying against the inside of the bone.

McBride and Turner[1] discuss the important question whether adenoids may be one of the ways by which tuberculosis enters into the system. Specimens from one hundred persons yielded three tuberculous ones, the criterion being "giant cells, with their marginal zone of nuclei, surrounded by endothelioid cells and areas of degeneration," but in no case were either bacilli or caseation detected. The results obtained by other observers give higher percentages, viz.: Pilliet, 7.5; Dieulafoy, 5.7 by histological examination, 20 by inoculation experiments; Brindel, 12.5; Gottstein, 12; Pluder and Fischer, 15.6; and the authors think that probably Dieulafoy's 20 per cent. is nearer the truth than their 3 per cent.

Provided no nasal injections are used after operation, otitis media is rare. Enlarged cervical glands sometimes disappear after removal of the growth. No good is to be expected in cases of deafness caused by sclerosis; but where the membranes are retracted and inflation benefits, the operation is of great value, also in otorrhœa.

Grant[2] considers that, as regards the recurrence or persistence of the symptoms of adenoids after removal, this is generally due to one or more of the following causes:—(1) The subsequent development of hypertrophy of the nasal mucous membrane; (2) Neglect on the part of the patient to keep up the practice of nasal breathing; (3) Anterior projection of the atlas vertebra; (4) Fixed idea.

Molinié[3] publishes three cases of **atrophic rhinitis** cured by the exclusive use of antitoxin injections, and Compaired calls attention to the resemblance between the ozœna microbe and the Klebs-Löffler bacillus, and states that he considers the antitoxin treatment the most certain one in this disease, the crusts

[1] *Edinb. M.J.*, 1897, N.S. i. 355, 471, 598; abstract in *J. Laryngol.*, 1897, xii. 393.

[2] *J. Laryngol.*, 1897, xii. 415.

[3] *Ann. d. Mal. de l'Oreille, du Larynx* [etc.], 1897, xxiii. 406; abstract in *J. Laryngol.*, 1897, xii. 395.

diminishing and the odour disappearing after the second or third injection of four to six cubic centimètres.

Middlemass Hunt[1] considers that we should submit patients who have **laryngeal growths** that are to be removed *per vias naturales* to some preliminary training. This consists in the introduction of probes into the larynx for a few days previous to operation. This was absolutely necessary in the pre-cocaine days, and all who have worked with Schroetter in Vienna in those days will remember, as I do, the thoroughness with which this was done. Hunt, whilst using 20 per cent. solutions of cocaine as a swab, trains his patients also.

If we use forceps with serrated edges to the blades and the growth does not come off readily, we ought to let it go and employ forceps with cutting edge, lest we tear away from the vocal cord the mucous membrane or even the cord itself, as Voltolini did. The snare is useful in growths in the anterior commissure and in multiple papillomata: it was easier to use this instrument before cocaine, as the vocal cords were approximated and the growth pushed into the loop; now they are flaccid, it is quite difficult to get hold of it. Hunt prefers the small cutting forceps of Schroetter, or the double curette of Krause to the large forceps of Mackenzie, which he considers unnecessarily powerful. The catheter curve to the forceps is better than the right-angled instrument, as the epiglottis is raised whilst the operation is being done, and so a better view of the larynx is obtained. Curettage or scraping is useful in diffuse multiple papillomata and in small warts in the anterior commissure. Knives, caustics, and the galvano-cautery are all to be used only with the greatest caution; and brushing with pure lactic acid, if a caustic is used, is as good as anything. In young children with multiple papillomata, Spicer's chloroform and cocaine anæsthesia and Kirschstein's direct method are both valuable.

Greville MacDonald[2] opened a very important discussion on **turbinotomy** at Montreal, which ought to be read carefully by everyone interested in nasal surgery. Many of us who saw the wonderful array of inferior turbinated bodies that were shown at the Bristol meeting of the British Medical Association were surprised that such a mutilating operation should be so frequently necessary. This discussion showed that few specialists practise it except in very rare cases, and it is evidently an operation to be avoided, for various reasons, except as a last resource.

BARCLAY J. BARON.

1 *J. Laryngol.*, 1897, xii. 420. 2 *Brit. M. J.*, 1897, ii. 1389.

